# Systems Pharmacology-Based Strategy to Investigate the Mechanism of Ruangan Lidan Decoction for Treatment of Hepatocellular Carcinoma

**DOI:** 10.1155/2022/2940654

**Published:** 2022-12-17

**Authors:** Cuilan Chen, Ting Liang, Qihui Wu, Zheyi Zhou, Mingmin Zhang, Dongshan Feng, Jingrui Tao, Tao Si, Min Cai

**Affiliations:** ^1^Department of Neurology, The Third Affiliated Hospital of Guangxi University of Chinese Medicine, Liuzhou Traditional Chinese Medical Hospital, Liuzhou 545000, China; ^2^Department of Oncology, The Third Affiliated Hospital of Guangxi University of Chinese Medicine, Liuzhou Traditional Chinese Medical Hospital, Liuzhou 545000, China; ^3^Clinical Research Center, Hainan Provincial Hospital of Traditional Chinese Medicine, Haikou 570100, China; ^4^Department of Spleen and Stomach Liver Diseases, Hainan Provincial Hospital of Traditional Chinese Medicine, Haikou 570100, China

## Abstract

epatocellular carcinoma (HCC) is one of the leading contributors to cancer mortality worldwide. Currently, the prevention and treatment of HCC remains a major challenge. As a traditional Chinese medicine (TCM) formula, Ruangan Lidan decoction (RGLD) has been proved to own the effect of relieving HCC symptoms. However, due to its biological effects and complex compositions, its underlying mechanism of actions (MOAs) have not been fully clarified yet. In this study, we proposed a pharmacological framework to systematically explore the MOAs of RGLD against HCC. We firstly integrated the active ingredients and potential targets of RGLD. We next highlighted 25 key targets that played vital roles in both RGLD and HCC disease via a protein-protein interaction (PPI) network and Kyoto Encyclopedia of Genes and Genomes (KEGG) pathway enrichment analyses. Furthermore, an ingredient-target network of RGLD consisting of 216 ingredients with 306 targets was constructed, and multilevel systems pharmacology analyses indicated that RGLD could act on multiple biological processes related to the pathogenesis of HCC, such as cellular response to hypoxia and cell proliferation. Additionally, integrated pathway analysis of RGLD uncovered that RGLD might treat HCC through regulating various pathways, including MAPK signaling pathway, PI3K/Akt signaling pathway, TNF signaling pathway, and ERBB signaling pathway. Survival analysis results showed that HCC patients with low expression of VEGFA, HIF1A, CASP8, and TOP2A were related with a higher survival rate than those with high expression, indicating the potential clinical significance for HCC. Finally, molecular docking results of core ingredients and targets further proved the feasibility of RGLD in the treatment of HCC. Overall, this study indicates that RGLD may treat HCC through multiple mechanisms, which also provides a potential paradigm to investigate the MOAs of TCM prescription.

## 1. Introduction

Hepatocellular carcinoma (HCC) is one of the pathological types of primary liver cancer, characterized by insidious onset, rapid progress, high mortality, and along with poor prognosis [1–3]. To date, the commonly used therapeutic drugs for HCC include sorafenib [4], cisplatin [5], and lenvatinib [6], which have been proved to treat HCC through proapoptotic, antiangiogenesis, and antiproliferation effects [7–9]. Despite that these drugs to some extent show therapeutic actions on HCC, the adverse reactions arisen by them also have been reported. For example, long-term exposure to sorafenib, cisplatin, and lenvatinib often lead to drug tolerance and disease progression from drug toxic effects [10, 11]. Therefore, it is an urgent need to search for safe and effective therapeutic drugs for HCC.

Traditional Chinese medicine (TCM) can prolong the survival period of patients with cancer via enhancing the body's immunity, as well as having the characteristics of low toxicity and reversal of multidrug resistance. It is also an important part of comprehensive treatment measures for malignant tumor [12–14]. Indeed, multiple TCM prescriptions have been demonstrated to treat HCC, including Xiao Chai Hu Tang (XCHT), Gan Lu Yin (GLY), and Wu Ling San (WLS) [15]. For instance, studies have revealed that XCHT is capable of inhibiting the development of HCC by suppressing the activation of stellate cells [16]. Ruangan Lidan decoction (RGLD), a herb combination based on XCHT, is composed of 11 Chinese herbs, including *Pinellia ternata (Thunb.)* Breit. (Ban Xia, BX), *Bupleurum chinense DC.* (Chai Hu, CH), *Sedum sarmentosum* Bunge. (Chui Pen Cao, CPC), *Glycyrrhiza uralensis* Fisch. (Gan Cao, GC), *Panax ginseng* C.A. Mey. (Hong Shen, HS), *Scutellaria baicalensis* Georgi. (Huang Qin, HQ), *Curcuma Longa* L. (Jiang Huang, JH), *Grangea maderaspatana* (Tian Ji Huang, TJH), *Prunella vulgaris* L. (Xia Ku Cao, XKC), *Corydalis yanhusuo* W.T. Wang (Yan Hu Suo, YHS), and *Artemisia capillaris* Thunb. (Yin Chen, YC). Our previous clinical studies have found that RGLD can effectively reduce transaminase, protect liver function, help prevent recurrence and metastasis of patients with HCC, and prolong the disease-free survival of HCC [17, 18]. In addition, it has been reported that RGLD can increase the levels of CD3^+^, CD4^+^, and CD4^+^/CD8^+^, improving patients' immune function and their quality of life after chemotherapy [19]. However, the MOAs of RGLD in treatment of HCC are still unclear.

System pharmacology is an emerging discipline that can describe the complex interactions between the drugs and biological systems, including the human body, organs, and diseases from the perspective of network. This approach is aimed at investigating the changes in the functions and reactions within the human body induced by drugs, thus providing new tools and strategies for the interpretation of complex disease mechanisms and modernization of TCM [20, 21]. Indeed, systems pharmacology has been widely applied to TCM field. For example, recent in vivo study has demonstrated that Elian granules may play a critical role to treat rat precancerous lesions of gastric cancer through the MAPK signaling pathway based on systems pharmacology approach [22].

**Figure 1:**
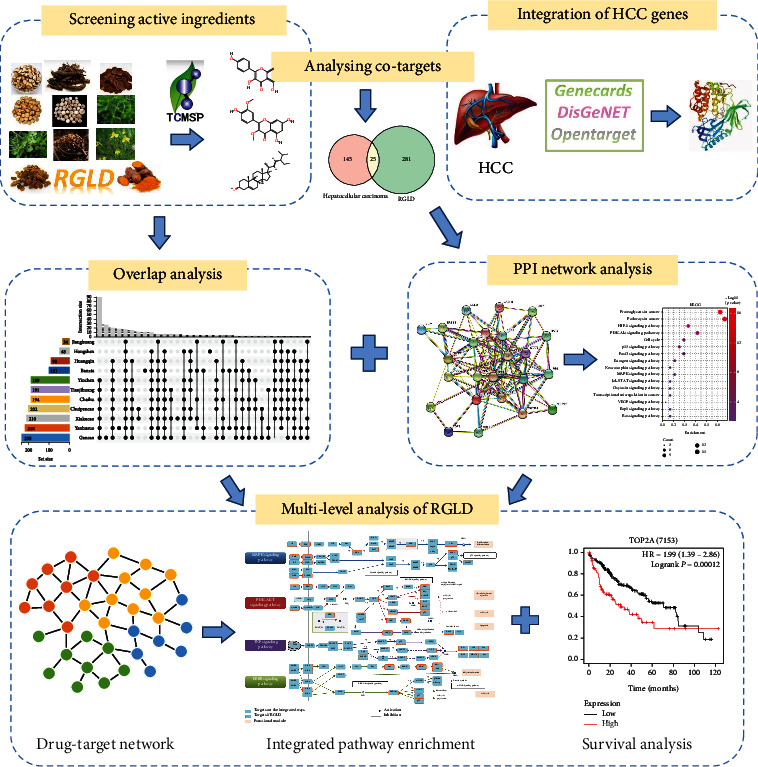
The flow chart of systems pharmacology approach for elucidating the MOAs of RGLD against HCC.

In the study, we attempted to establish a complete pharmacological framework to systematically explore the molecular MOAs of RGLD against HCC (Figure 1). Specifically, we firstly collected the chemical ingredients of RGLD from the Traditional Chinese Medicine Systems Pharmacology (TCMSP) database and integrated HCC disease genes from GeneCards, DisGeNET, and OpenTarget databases. Subsequently, we performed the PPI analysis and the Kyoto Encyclopedia of Genes and Genomes (KEGG) pathway annotation on overlapped targets. We next constructed a drug-target network for RGLD based on the herbal compositions and protein targets. Furthermore, we also made multilevel systems pharmacology analysis, including biological process (BP) analysis, molecular function (MF) analysis, cellular component (CC) analysis, and integrated pathway to elucidate the possible mechanisms of RGLD against HCC. Finally, we performed the overall survival analysis of 25 overlapping targets and highlighted some key proteins in RGLD for molecular docking.

## 2. Material and Methods

### 2.1. Active Ingredient Screening

TCMSP (http://tcmspw.com/tcmsp.php) database was used to collect the effective components and related targets of the 11 herbs in RGLD. The screening criteria were oral bioavailability (OB) > 30% and drug-likeness (DL) > 0.18 [23, 24], since both of them played important roles in evaluating the activity of drugs. Finally, 216 active ingredients in RGLD were obtained in total. The corresponding number of the obtained ingredients in BX, CH, CPC, GC, HS, HQ, JH, TJH, XKC, YHS, and YC is 13, 17, 5, 92, 4, 36, 3, 8, 11, 49, and 13, respectively. The detailed information of herbal ingredients was shown in Table S1.

### 2.2. Manual Curation of HCC Disease Genes

Using “Hepatocellular Carcinoma” as the key word, we screened the disease genes of HCC from GeneCards database (https://www.genecards.org/), DisGeNET database (http://www.disgenet.org/), and OpenTarget database (https://platform.opentargets.org/) [25, 26]. The screening criteria for each database were set to score greater than 30, 0.03, and 0.35, respectively.

### 2.3. PPI Network Construction

The intersection targets of drug-target-disease genes were imported into the STRING database (https://string-db.org/) to construct a PPI network [27]. The degree centrality is considered as the main parameter, and the core proteins are determined by combining compactness centrality and intermediate centrality [28].

### 2.4. Go Oncology (GO) Enrichment Analysis and KEGG Pathway Enrichment

DAVID database (https://david.ncifcrf.gov/home.jsp) was used to perform GO terms for further exploring the potential mechanism of RGLD against HCC, including BP analysis, MF analysis, and CC analysis. All gene names were entered into the DAVID database and limited the species to *Homo sapiens*. Only those terms with the *P* value < 0.05 were retained [29].

### 2.5. Drug-Target Network Construction

Drug-target interactions were obtained from the TCMSP database, which were integrated based on two sources: experimental validation and predicted drug-target pairs [30]. Cytoscape 3.8.0 was utilized to construct the drug-target network of RGLD. In graphical network, compounds or genes were presented by nodes while interactions were encoded by edges. The degree of each node was calculated, which represents the number of edges linked to it, characterizing the most important nodes in a network. The more edges, the more connections the nodes have, and the bigger the nodes are.

### 2.6. Survival Analysis

We imported overlapped targets of drugs and diseases to the Kaplan-Meier plotter (http://kmplot.com/) to analyze the correlation between the expression of crucial anti-HCC targets in RGLD and the survival rate of HCC patients. The targets with *P* value < 0.05 were selected [31]. Furthermore, targets with low risk and long survival were screened according to the median survival.

### 2.7. Molecular Docking

The molecular docking analysis was performed on AutoDock Vina and AutoDock Tools. Targets of HCC and 2D structures of the active ingredients of RGLD were downloaded from the Protein Data Bank (PDB) database (http://www.rcsb.org/) and PubChem (https://pubchem.ncbi.nlm.nih.gov/) [32, 33]. The AutoDock Tools was used to hydrogenate the protein, specify the bond level, remove solvents, define the grid box, and generate the receptor grid file while the AutoDock Vina was used to dock between the ligand structure and the generated receptor grid file. The results were subsequently opened with PyMOL and LigPlus for previewing, analyzing, and graphing. Generally, the lower the binding energy is, the better the docking effect is.

## 3. Results

### 3.1. Overlap Analysis of Herbal Components and Targets of RGLD

**Figure 2:**
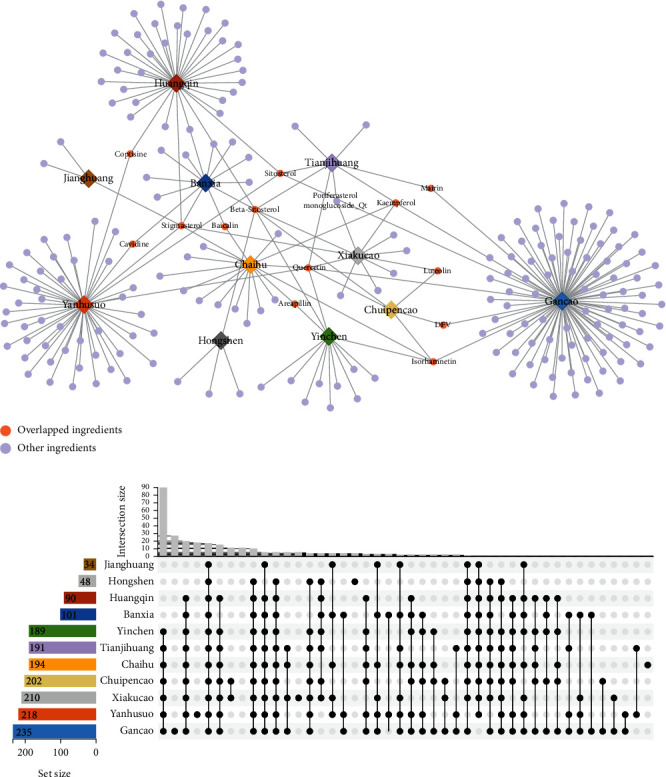
Overlap analysis of herbal ingredients (a) and corresponding targets (b) of RGLD. The corresponding number of ingredients for each herb is 3 (JH), 4 (HS), 36 (HQ), 13 (BX), 13 (YC), 8 (TJH), 17 (CH), 5 (CPC), 11 (XKC), 49 (YHS), and 92 (GC). The relative number of targets in RGLD for each herb is 34 (JH), 48 (HS), 90 (HQ), 101 (BX), 189 (YC), 191 (TJH), 194 (CH), 202 (CPC), 210 (XKC), 218 (YHS), and 235 (GC).

As a key theory of TCM, “Jun-Chen-Zuo-Shi” has guided physicians to formulate herbal medicines for over thousands of years [34]. This principle indicates the different roles played by each herb in a prescription and its change rule of drug efficacy after compatibility. To clarify the potential formula principle of RGLD in treating HCC at a molecular level, we performed overlap analysis of these herbal components and their relative targets. As shown in Figure 2(a), multiple herbs share overlapped ingredients. Among the 11 Chinese herbs in RGLD, TJH and XKC own the highest number of common ingredients (*n* = 4), including beta-sitosterol, kaempferol, poriferasterol monoglucoside_qt, and quercetin. Beta-sitosterol has been reported to exhibit ameliorative effects on HCC due to its antioxidant activities [35]. Kaempferol also has strong anticancer activities and can inhibit the invasive properties of HCC by targeting MMP-9 and Akt pathways [36]. In addition, quercetin also has been confirmed to inhibit growth of HCC by apoptosis induction [37]. Interestingly, we found that six herb-herb pairs all have three common ingredients, suggesting their intrinsic interactions. The six herb-herb pairs are CPC and GC, CPC and XKC, CPC and YC, BX and HQ, CH and XKC, and CH and YC. Meanwhile, the 11 herbs in RGLD could act on 306 targets (Figure 2(b)). Among them, GC and YHS have the largest number of overlapped targets (*n* = 191), which implies that they may play synergistic effects on HCC. These targets include PGR, PTGS1, NCOA2, progesterone receptor, prostaglandin G/H synthase 2, and beta-1 adrenergic receptor. Studies have deciphered that NCOA2 is an important tumor suppressor gene, and activation of NCOA2 is beneficial to inhibit the occurrence and development of HCC [38]. Taken together, RGLD may amplify its therapeutic effect by modulating these common targets.

### 3.2. Overlap Analysis of Ingredient Targets and Disease Genes

**Figure 3:**
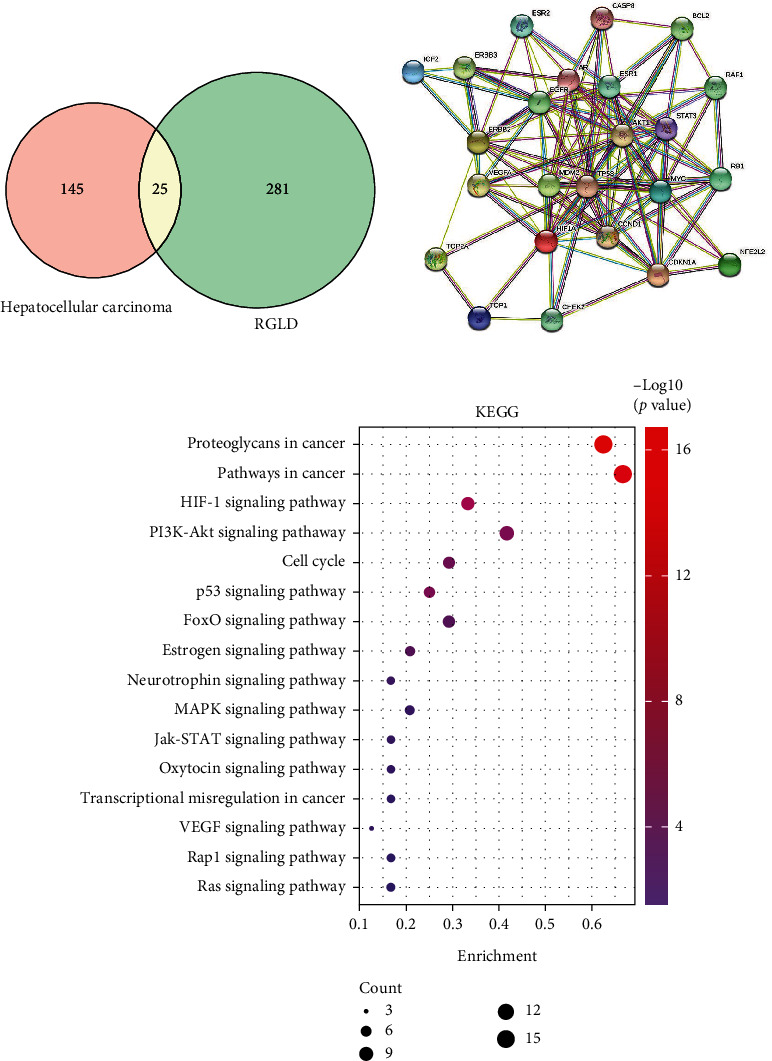
Overlap analysis of disease genes and herbal targets. Overlapped genes (a), PPI network analysis (b), and KEGG pathway enrichment analysis (c).

We acquired 170 HCC genes from DisGeNET, OpenTarget, and GeneCards databases after removing duplicated genes. The detailed information of the 170 disease genes were provided in Table S2. We next performed overlap analysis on disease genes and herbal targets by Venny, and 25 intersection targets of disease and herbs were obtained (Figure 3(a)). Subsequently, we imported these 25 overlapped targets into the STRING database to construct PPI network with a high confidence of >0.7. Topological analysis of PPI network indicated that TP53, STAT3, MDM2, HIF1A, ESR1, ERBB3, EGFR, CDKN1A, and CCND1 were the key anti-HCC targets in RGLD (Figure 3(b)) [39].

We further performed KEGG pathway enrichment analysis of the 25 intersection targets by using DAVID online platform. As displayed in Figure 3(c), these targets were mainly involved in hypoxia-inducible factor-1 (HIF-1) signaling pathway, p53 signaling pathway, estrogen signaling pathway, etc. Multiple evidences have confirmed that they are highly related with HCC. For example, HIF-1 has been demonstrated to be associated with the prognosis of HCC [40]. p53 signaling pathway is a significant apoptotic target in many cancer types. It has been proved that activation of p53 signaling pathway can control proliferation and trigger apoptosis of HCC cells [41, 42]. Furthermore, estrogen signaling pathway was reported to protect the occurrence and development of HCC. Recent studies have also showed that estrogen signaling pathway can regulate miRNAs and the microenvironment of HCC to exert its anti-HCC effect [43, 44].

### 3.3. Drug-Target Network

**Figure 4:**
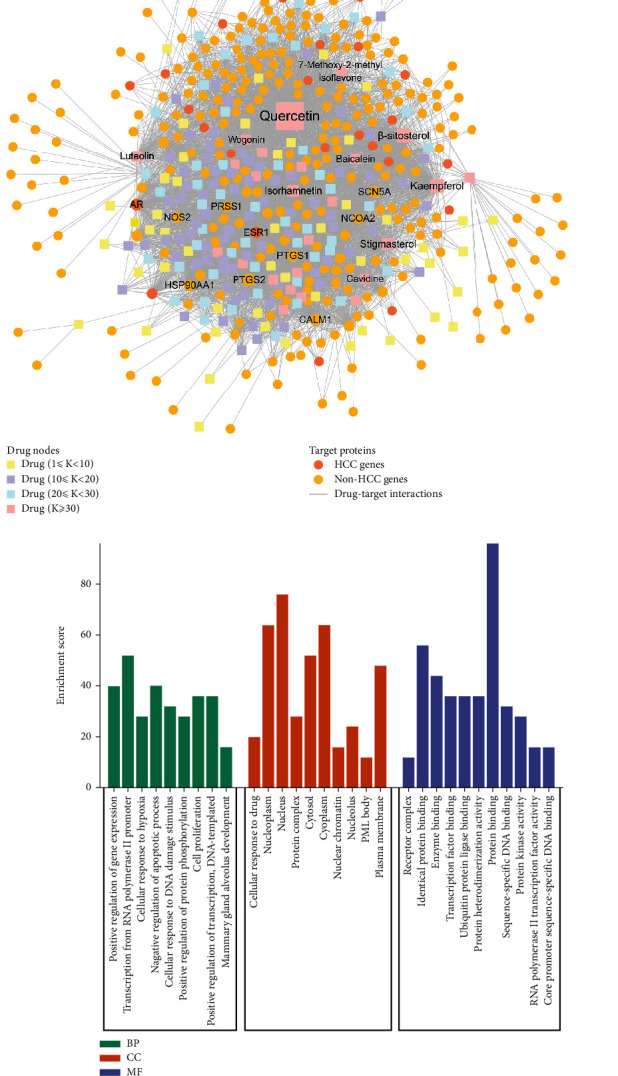
Drug-target network (a) and GO enrichment analysis (b) of RGLD.

To construct a drug-target network for RGLD, we integrated the herbal ingredients of RGLD and its relative targets. A total of 5320 drug-target interactions (DTIs) were obtained in the network, connecting 216 ingredients with 306 targets. Interestingly, we found that these 306 targets were composed of 25 HCC targets and 281 non-HCC targets. Since TCM has the characteristics of multitarget pharmacological action, it can not only act on disease genes directly but also exert the therapeutic effect of HCC by acting on other related genes [24, 45].  Figure 4(a) implies that most compounds are intensively linked to multiple targets. Among the 216 ingredients, the top 10 with the largest target degree (*D*) includes quercetin (*D* = 1063), beta-sitosterol (*D* = 277), kaempferol (*D* = 252), stigmasterol (*D* = 186), isorhamnetin (*D* = 148), luteolin (*D* = 114), baicalein (*D* = 74), cavidine (*D* = 56), wogonin (*D* = 45), and 7-methoxy-2-methyl isoflavone (*D* = 43). Emerging evidence shows that these components are highly related to HCC. For example, baicalein can suppress HCC cell growth and survival through downregulation of CD24 [46]. Moreover, luteolin exerts anti-HCC activity and promotes cell apoptosis by activating casepase-3, increasing Bax protein expression and decreasing Bcl-2 level [47]. In addition, lots of proteins are also targeted by various components. Among them, HSP90AA1 (*K* = 155) has the highest compound connections, followed by CALM1 (*K* = 141) and ESR1 (*K* = 105). As a family member of heat-shock proteins (HSPs), HSP90AA1 is a potential diagnostic and prognostic biomarker and therapeutic target for HCC, acting as an active role in tumor and immune-related signaling pathways [48, 49]. CALM1 also has been reported to be closely associated with HCC. CALM1 gene decreases gradually with tumor enlargement and is also a potential biomarker for the diagnosis of HCC [50]. Moreover, high expression of ESR1 has a protective effect on the overall survival of HCC and is associated with a favorable prognosis [51, 52].

We next performed GO enrichment analysis on the top 30 targets in the drug-target network. As shown in Figure 4(b), the GO enrichment results include 9 BP, 10 CC, and 11 MF items. Most of the BP terms are associated with HCC, such as cellular response to hypoxia and cell proliferation. Previous literature has proved that HCC is the most serious hypoxic tumor, which can induce an increase malignancy and poor prognosis of HCC [53–55]. Additionally, hypoxia also causes metabolic changes through HIF and improves antitumor drug resistance. Therefore, overcoming hypoxia is a significant strategy for the treatment of HCC [56]. A growing body of evidence suggests that inhibition of cell proliferation is a significant means to treat HCC and beneficial for improving patient survival [57]. Similarly, the CC terms (e.g., cellular response to drug) also exerts an important effect in the pathogenesis of HCC. For instance, drug resistance is one of the cellular responses to drugs. Studies have shown that HCC resistance to chemotherapeutic drugs is a key limitation of curative treatment and the cause of treatment failure and recurrence [58]. Multiple MF terms also participate in the pathogenesis of HCC. Transcription factor, such as KLF9, was reported to play a critical role in HCC development, which could significantly suppress the growth of HCC in vivo [59]. Moreover, protein kinase activity (e.g., mitogen-activated protein kinase activity), has been proved to inhibit the proliferation of HCC [60]. Taken together, the GO enrichment analysis shows that RGLD could act on multiple BPs, CCs, and MFs to exert therapeutic effects on HCC.

### 3.4. Integrated Pathway Analysis of RGLD on HCC

**Figure 5:**
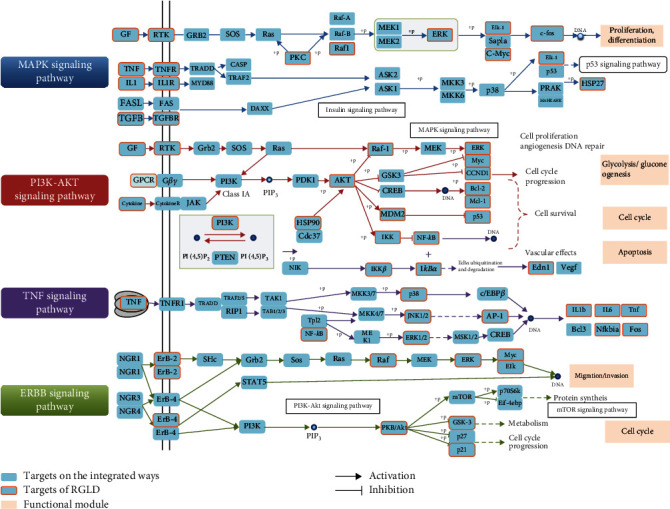
The functional modules and integrated pathways based on HCC. The dark blue nodes denote protein targets of RGLD and the light blue nodes indicate targets on the integrated pathway. The yellow nodes represent functional modules.

In order to construct an HCC-integrated pathway, targets of RGLD were mapped into these pathways associated with the pathological process of HCC. As depicted in Figure 5, several functional modules, such as cell proliferation, cell cycle, and apoptosis, play roles in the integrated pathways. Here, we selected four representative modules to uncover the potential therapeutic mechanisms of RGLD towards HCC.

#### 3.4.1. Cell Proliferation and Apoptosis Module

Proliferation and apoptosis of cells have been considered as a contributing factor in the occurrence, development, and prognosis of HCC [61]. Abnormal proliferation and survival of HCC cells can lead to progression of this disease [62]. MAPK signaling pathway has been confirmed to regulate various cellular activities in HCC, including differentiation, proliferation, apoptosis, and inflammation [63]. Increasing evidence indicates that suppressing the expression of MAPK signaling pathway or its related proteins can effectively inhibit HCC cell proliferation and promote HCC cell apoptosis [64–66]. As displayed in Figure 5, RGLD could act on multiple targets on MAPK signaling pathways, such as PKC and ERK, which indicates the potential role of MAPK signaling pathway for HCC prevention and treatment.

#### 3.4.2. Autophagy Module

Autophagy is a lysosomal-dependent catabolic pathway, which is closely related to the pathogenesis of HCC [67]. Indeed, autophagy can support the proliferation and metastasis of HCC, whereas it can also remove toxic components of tumors to inhibit tumor [68–70]. Phosphatidylinositol 3-kinase (PI3K)/protein kinase B (Akt) signaling pathway is closely related to autophagy [71]. Previous studies implied that autophagy could be induced by downregulating the PI3K/Akt signaling pathway, thus restraining the progress of HCC [72]. Figure 5 indicates that a large number of compounds in RGLD could act on the targets of PI3K/Akt signaling pathway, suggesting the potential therapeutic mechanism of RGLD against HCC.

#### 3.4.3. Inflammation Module

It is acknowledged that persistent chronic inflammation is a cancer promoter while HCC is one of the most common inflammation-related cancers [73]. The occurrence and development of HCC have been proved to be inflammation-related carcinogenic processes, and inhibition of inflammation may be an important measure to prevent and treat HCC [58]. Tumor necrosis factor (TNF) signaling pathway is associated with cellular inflammation, death, or survival [74]. Previous literature indicates that inflammatory factors, such as TNF-*α*, can promote tumor growth and poor prognosis of HCC [75]. Additionally, inflammation can promote malignant progression of cancers, and the use of anti-inflammatory drugs is capable to reduce the risk of HCC development and improve the survival rate of HCC [76, 77]. As exhibited in Figure 5, the protein targets of RGLD are enriched in the TNF signaling pathway, indicating that RGLD may play roles in inflammatory response of HCC.

#### 3.4.4. Cell Migration and Invasion Module

The epidermal growth factor receptor (ERBB) signaling pathway is a transmembrane receptor tyrosine kinase, which plays a significant role in HCC, such as controlling cell migration and invasion [78, 79]. Recent studies have confirmed that inhibiting the expression of ERBB signaling pathway or signal transduction of ERBB-related family proteins is beneficial to suppress cell migration and invasion of liver cancer [80, 81]. Figure 5 shows that RGLD could act on several critical proteins in ERBB signaling pathway, suggesting that RGLD may participate in the occurrence and development of HCC through ERBB signaling pathway.

### 3.5. Survival Analysis

**Figure 6:**
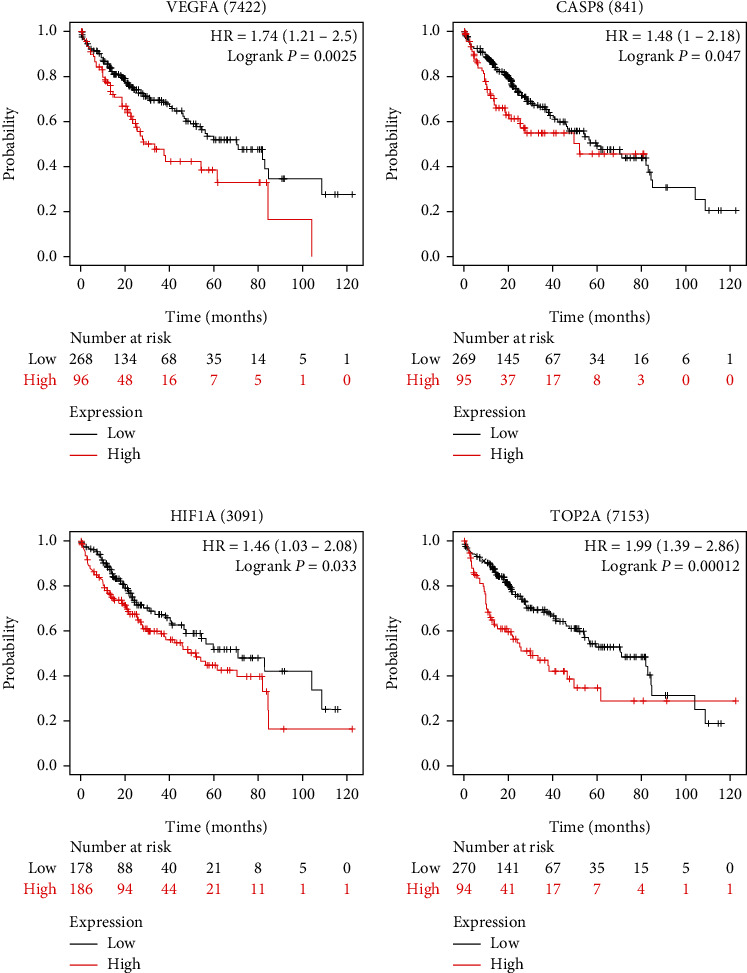
Overall survival analyses for VEGFA (a), CASP8 (b), HIF1A (c), and TOP2A (d).

In order to evaluate the potential clinical significance of these targets, we performed survival analysis of 25 overlapped targets in RGLD and HCC by using the Kaplan-Meier plotter. As shown in Figure 6(a)–6(d), patients with low expression of VEGFA (a ), CASP8 (b ), HIF1A (c ), and TOP2A (d) were related with a higher survival rate than those with high expression, which conforms to previous literature evidence. For example, type IIA topoisomerase (TOP2A) has been confirmed to be highly expressed in HCC, and its expression is positively related to poor prognosis [82]. Knocking down the expression of TOP2A can curb the metastasis proliferation and invasion of HCC and improve the survival rate [83]. Therefore, we deduced that the expression levels of VEGFA, HIF1A, CASP8, and TOP2A played significant roles in the pathogenesis of HCC and were correlated with the clinical prognosis of HCC.

### 3.6. Molecular Docking Analysis

**Figure 7:**
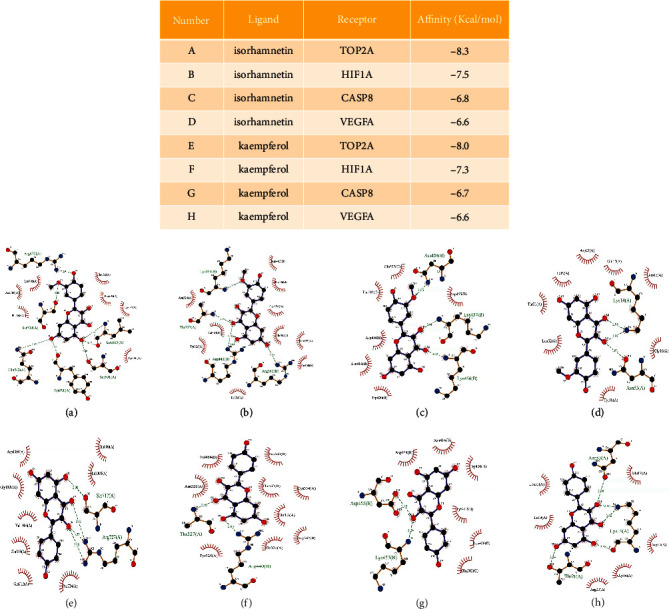
Small molecules with core protein docking. In the table, number is the order of molecular docking graph. Ligand is drug small molecule. Receptor is drug target. Affinity (Kcal/mol) is docking energy. In the molecular docking diagram, red, blue, black, and green represent oxygen, nitrogen, carbon, and residues, respectively. Furthermore, the shape of the eyelash represents the surrounding residue, and the number represents the bond length. (a) Isorhamnetin: TOP2A, (b) isorhamnetin: HIF1A, (c) isorhamnetin: CASP8, (d) isorhamnetin: VEGFA, (e) kaempferol: TOP2A, (f) kaempferol: HIF1A, (g) kaempferol: CASP8, and (h) kaempferol: VEGFA.

To explore the feasibility of RGLD in the treatment of HCC, molecular docking analysis was performed on the core active ingredients of HCC (quercetin, beta-sitosterol, kaempferol, stigmasterol, and isorhamnetin) and the key targets of HCC (VEGFA, HIF1A, CASP8, and TOP2A). The PDB numbers of the crystal structures of VEGFA, HIF1A, CASP8, and TOP2A were 3QTK, 4H6J, 1QTN, and 4MF9, respectively. In Figures 7(a)–7(h), after molecular docking analyses of drug components and disease genes, we found that isorhamnetin and four disease genes (Figures 7(a)–7(d)) and kaempferol and four disease genes (Figures 7(e)–7(h)) that docking effect was better. Among them, isorhamnetin had the lowest docking scores (affinity = −8:3) with the four disease genes, indicating its good docking effect. The docking diagrams with four protein macromolecules were also shown in Figures 7(a)–7(h). Taking isorhamnetin for example, its hydrogen bond lengths with CASP8 Asn458(B) residue, Lys457(B), and Lys456(B) residue were 3.06 angstroms, 3.04 angstroms, and 3.02 angstroms, respectively.

## 4. Discussion

HCC is the most common type of liver cancer and also the second leading cause of cancer-correlated mortality worldwide [84]. Surgical resection followed by chemotherapy is the most common curative treatment scheme for HCC. However, drug resistance, toxic, and side effects can be clearly observed after chemotherapy. Various chemotherapeutic agents, such as cisplatin and sorafenib, demonstrate lower efficacy in HCC than in other cancers [58]. Therefore, it is necessary to find a more safe and effective anti-HCC therapy. With a long history, Chinese herbal medicines have been proved to be beneficial for the prevention and treatment of HCC [85, 86]. RGLD was demonstrated to inhibit tumor growth, enhance the body immunity, and prolong disease-free survival of patients during the treatment of HCC [17, 19]. However, the specific pharmacological MOAs of RGLD against HCC have not been fully elaborated.

In this study, we firstly performed overlap analysis on herbal ingredients and potential targets to investigate the rationality of RGLD. We next integrated the PPI network of 25 hub targets and performed KEGG pathway enrichment analysis. We further constructed a drug-target network of RGLD and proposed multilevel analysis to explore the potential mechanism of RGLD in the treatment of HCC through GO term annotations, HCC-integrated pathway analysis, and survival analysis. According to the systems pharmacology results, RGLD may play a reliable intervention effect on HCC through multipathway, multitarget, and multimechanism. For example, the targets of RGLD are enriched in PI3K/Akt pathways, ERBB pathways, TNF pathways, MAPK pathways, etc. (Figure 5). These pathways are involved in inflammatory response, cell proliferation, metastasis, autophagy, and apoptosis of HCC [87, 88]. Recent studies have revealed that the extract of RGLD significantly inhibited the proliferation, migration of HCC, and induced apoptosis [89, 90]. Hernández-Aquino et al. [91] also found that naringin, one of the main active components of RGLD decoction, has anti-inflammatory and antitumor effects on HCC by regulating MAPK signal pathway, which further validated our predictive results. Survival analysis indicates that VEGFA plays a vital role in HCC, and emerging evidence has confirmed that inhibition of VEGFA expression can inhibit HCC cell migration and proliferation [92, 93]. Moreover, molecular docking further proved the feasibility of RGLD in the treatment of HCC. This study preliminarily deciphered the potential MOAs of RGLD in the treatment of HCC which suggests that systems pharmacology could serve as a valid approach to investigate the underlying MOAs of TCM formula against complex diseases.

Although the current drug compositions and dose of RGLD showed efficacy on HCC patients, there were still several limitations in our study. Firstly, due to the complexity of HCC disease and the intrinsic interactions and multiple complicated ingredients in the mixture of different plants, thus, our study could not entirely reflect the actual function of different drug dosages of RGLD in the human body. Secondly, the systems pharmacology results are based on the interaction between components and drug targets of medicinal materials, which rarely depend on the drug dosages. Therefore, the current study does not consider the effect of different drug dosages on systems pharmacology results. Collectively, it is necessary to further explore the possible mechanism and the most suitable dose of RGLD in the treatment of HCC in future clinical and experimental studies.

## Data Availability

The datasets generated for this study are available upon request from the corresponding authors.

## Supplementary Materials

Supplementary 1 Supplementary Table S1: the detailed information of 216 herbal ingredients of RGLD.

Supplementary 2 Supplementary Table S2: the detailed information of the 170 disease genes.
