# Supplementary material for: Systems Pharmacology-Based Strategy to Investigate the Mechanism of Ruangan Lidan Decoction for Treatment of Hepatocellular Carcinoma
Source: Comput Math Methods Med. 2022 Dec 17;2022:2940654. doi: 10.1155/2022/2940654 (PMC9791079; doi:10.1155/2022/2940654)
Supplement: Supplementary 1 — Supplementary Table S1: the detailed information of 216 herbal ingredients of RGLD. [file 2940654.f1.pdf]

Name	Mol ID	Molecule Name
Banxia	MOL001755	24-Ethylcholest-4-en-3-one
Banxia	MOL002670	Cavidine
Banxia	MOL002714	baicalein
Banxia	MOL002776	Baicalin
Banxia	MOL000358	beta-sitosterol
Banxia	MOL000449	Stigmasterol
Banxia	MOL005030	gondoic acid
Banxia	MOL000519	coniferin
Banxia	MOL006936	10,13-eicosadienoic
Banxia	MOL006937	12,13-epoxy-9-hydroxynonadeca-7,10-dienoic acid
Banxia	MOL006957	(3S,6S)-3-(benzyl)-6-(4-hydroxybenzyl)piperazine-2,5-quinone
Banxia	MOL003578	Cycloartenol
Banxia	MOL006967	beta-D-Ribofuranoside, xanthine-9
Chaihu	MOL001645	Linoleyl acetate
Chaihu	MOL002776	Baicalin
Chaihu	MOL000449	Stigmasterol
Chaihu	MOL000354	isorhamnetin
Chaihu	MOL000422	kaempferol
Chaihu	MOL004598	3,5,6,7-tetramethoxy-2-(3,4,5-trimethoxyphenyl)chromone
Chaihu	MOL004609	Areapillin
Chaihu	MOL013187	Cubebin
Chaihu	MOL004	Longikaurin A

	624	
Chaihu	MOL004628	Octalupine
Chaihu	MOL004644	Sainfuran
Chaihu	MOL004648	Troxerutin
Chaihu	MOL004653	(+)-Anomalin
Chaihu	MOL004702	saikosaponin c_qt
Chaihu	MOL004718	α-spinasterol
Chaihu	MOL000490	petunidin
Chaihu	MOL000098	quercetin
Chuipencao	MOL001792	DFV
Chuipencao	MOL000354	isorhamnetin
Chuipencao	MOL000358	beta-sitosterol
Chuipencao	MOL000006	luteolin
Chuipencao	MOL000098	quercetin
Gancao	MOL001484	Inermine
Gancao	MOL001792	DFV
Gancao	MOL000211	Mairin
Gancao	MOL002311	Glycyrol
Gancao	MOL000239	Jaranol
Gancao	MOL002565	Medicarpin
Gancao	MOL000354	isorhamnetin
Gancao	MOL000359	sitosterol
Ganca	MOL003	Lupiwighteone

o	656	
Gancao	MOL003896	7-Methoxy-2-methyl isoflavone
Gancao	MOL000392	formononetin
Gancao	MOL000417	Calycosin
Gancao	MOL000422	kaempferol
Gancao	MOL004328	naringenin
Gancao	MOL004805	(2S)-2-[4-hydroxy-3-(3-methylbut-2-enyl)phenyl]-8,8-dimethyl-2,3-dihydropyrano[2,3-f]chromen-4-one
Gancao	MOL004806	euchrenone
Gancao	MOL004808	glyasperin B
Gancao	MOL004810	glyasperin F
Gancao	MOL004811	Glyasperin C
Gancao	MOL004814	Isotrifoliol
Gancao	MOL004815	(E)-1-(2,4-dihydroxyphenyl)-3-(2,2-dimethylchromen-6-yl)prop-2-en-1-one
Gancao	MOL004820	kanzonols W
Gancao	MOL004824	(2S)-6-(2,4-dihydroxyphenyl)-2-(2-hydroxypropan-2-yl)-4-methoxy-2,3-dihydrofuro[3,2-g]chromen-7-one
Gancao	MOL004827	Semilicoisoflavone B
Gancao	MOL004828	Glepidotin A
Gancao	MOL004829	Glepidotin B
Gancao	MOL004833	Phaseolinisoflavan
Gancao	MOL004835	Glypallichalcone
Gancao	MOL004838	8-(6-hydroxy-2-benzofuranyl)-2,2-dimethyl-5-chromenol
Gancao	MOL004841	Licochalcone B
Ganca	MOL004	licochalcone G

o	848	
Gancao	MOL004849	3-(2,4-dihydroxyphenyl)-8-(1,1-dimethylprop-2-enyl)-7-hydroxy-5-methoxy-coumarin
Gancao	MOL004855	Licoricone
Gancao	MOL004856	Gancaonin A
Gancao	MOL004857	Gancaonin B
Gancao	MOL004860	licorice glycoside E
Gancao	MOL004863	3-(3,4-dihydroxyphenyl)-5,7-dihydroxy-8-(3-methylbut-2-enyl)chromone
Gancao	MOL004864	5,7-dihydroxy-3-(4-methoxyphenyl)-8-(3-methylbut-2-enyl)chromone
Gancao	MOL004866	2-(3,4-dihydroxyphenyl)-5,7-dihydroxy-6-(3-methylbut-2-enyl)chromone
Gancao	MOL004879	Glycyrin
Gancao	MOL004882	Licocoumarone
Gancao	MOL004883	Licoisoflavone
Gancao	MOL004884	Licoisoflavone B
Gancao	MOL004885	licoisoflavanone
Gancao	MOL004891	shinpterocarpin
Gancao	MOL004898	(E)-3-[3,4-dihydroxy-5-(3-methylbut-2-enyl)phenyl]-1-(2,4-dihydroxyphenyl)prop-2-en-1-one
Gancao	MOL004903	liquiritin
Gancao	MOL004904	licopyranocoumarin
Gancao	MOL004905	3,22-Dihydroxy-11-oxo-delta(12)-oleanene-27-alpha-methoxycarbonyl-29-oic acid
Gancao	MOL004907	Glyzaglabrin
Gancao	MOL004908	Glabridin
Gancao	MOL004910	Glabranin
Ganca	MOL004	Glabrene

o	911	
Gancao	MOL004912	Glabrone
Gancao	MOL004913	1,3-dihydroxy-9-methoxy-6-benzofurano[3,2-c]chromenone
Gancao	MOL004914	1,3-dihydroxy-8,9-dimethoxy-6-benzofurano[3,2-c]chromenone
Gancao	MOL004915	Eurycarpin A
Gancao	MOL004917	glycyroside
Gancao	MOL004924	(-)-Medicocarpin
Gancao	MOL004935	Sigmoidin-B
Gancao	MOL004941	(2R)-7-hydroxy-2-(4-hydroxyphenyl)chroman-4-one
Gancao	MOL004945	(2S)-7-hydroxy-2-(4-hydroxyphenyl)-8-(3-methylbut-2-enyl)chroman-4-one
Gancao	MOL004948	Isoglycyrol
Gancao	MOL004949	Isolicoflavonol
Gancao	MOL004957	HMO
Gancao	MOL004959	1-Methoxyphaseollidin
Gancao	MOL004961	Quercetin der.
Gancao	MOL004966	3'-Hydroxy-4'-O-Methylglabridin
Gancao	MOL000497	licochalcone a
Gancao	MOL004974	3'-Methoxyglabridin
Gancao	MOL004978	2-[(3R)-8,8-dimethyl-3,4-dihydro-2H-pyrano[6,5-f]chromen-3-yl]-5-methoxyphenol
Gancao	MOL004980	Inflacoumarin A
Gancao	MOL004985	icos-5-enoic acid
Gancao	MOL004988	Kanzonol F
Ganca	MOL004	6-prenylated eriodictyol

o	989	
Ganca o	MOL004 990	7,2',4'-trihydroxy－5-methoxy-3－arylcoumarin
Ganca o	MOL004 991	7-Acetoxy-2-methylisoflavone
Ganca o	MOL004 993	8-prenylated eriodictyol
Ganca o	MOL004 996	gadelaidic acid
Ganca o	MOL000 500	Vestitol
Ganca o	MOL005 000	Gancaonin G
Ganca o	MOL005 001	Gancaonin H
Ganca o	MOL005 003	Licoagrocarpin
Ganca o	MOL005 007	Glyasperins M
Ganca o	MOL005 008	Glycyrrhiza flavonol A
Ganca o	MOL005 012	Licoagroisoflavone
Ganca o	MOL005 013	18α-hydroxyglycyrrhetic acid
Ganca o	MOL005 016	Odoratin
Ganca o	MOL005 017	Phaseol
Ganca o	MOL005 018	Xambioona
Ganca o	MOL005 020	dehydroglyasperins C
Ganca o	MOL000 098	quercetin
Hongs hen	MOL002 032	DNOP
Hongs hen	MOL000 358	beta-sitosterol
Hongs hen	MOL005 344	ginsenoside rh2
Hongs hen	MOL002 372	(6Z,10E,14E,18E)-2,6,10,15,19,23-hexamethyltetracosa-2,6,10,14,18,22-hexaene
Huang	MOL002	NEOBAICALEIN

qin	934	
Huang	MOL002	Panicolin
qin	932	
Huang	MOL012	5,7,4'-trihydroxy-8-methoxyflavanone
qin	246	
Huang	MOL002	Skullcapflavone II
qin	927	
Huang	MOL002	2,6,2',4'-tetrahydroxy-6'-methoxychaleone
qin	911	
Huang	MOL002	DIHYDROOROXYLIN
qin	937	
Huang	MOL000	(2R)-7-hydroxy-5-methoxy-2-phenylchroman-4-one
qin	228	
Huang	MOL002	Salvigenin
qin	915	
Huang	MOL000	ent-Epicatechin
qin	073	
Huang	MOL002	5,2',6'-Trihydroxy-7,8-dimethoxyflavone
qin	917	
Huang	MOL008	Moslosooflavone
qin	206	
Huang	MOL000	Stigmasterol
qin	449	
Huang	MOL001	bis[(2S)-2-ethylhexyl] benzene-1,2-dicarboxylate
qin	490	
Huang	MOL002	Diop
qin	879	
Huang	MOL002	epiberberine
qin	897	
Huang	MOL002	oroxylin a
qin	928	
Huang	MOL002	Eriodyctiol (flavanone)
qin	914	
Huang	MOL002	Carthamidin
qin	910	
Huang	MOL002	Dihydrobaicalin_qt
qin	913	
Huang	MOL000	Norwogonin
qin	525	
Huang	MOL010	11,13-Eicosadienoic acid, methyl ester
qin	415	
Huang	MOL002	dihydrooroxylin A
qin	926	
Huang	MOL012	rivularin

qin	266	
Huangqin	MOL002925	5,7,2',6'-Tetrahydroxyflavone
Huangqin	MOL002908	5,8,2'-Trihydroxy-7-methoxyflavone
Huangqin	MOL000358	beta-sitosterol
Huangqin	MOL000359	sitosterol
Huangqin	MOL012245	5,7,4'-trihydroxy-6-methoxyflavanone
Huangqin	MOL002933	5,7,4'-Trihydroxy-8-methoxyflavone
Huangqin	MOL001689	acacetin
Huangqin	MOL002909	5,7,2,5-tetrahydroxy-8,6-dimethoxyflavone
Huangqin	MOL001506	Supraene
Huangqin	MOL002714	baicalein
Huangqin	MOL000552	5,2'-Dihydroxy-6,7,8-trimethoxyflavone
Huangqin	MOL000173	wogonin
Huangqin	MOL001458	coptisine
Jianghuang	MOL000449	Stigmasterol
Jianghuang	MOL000493	campesterol
Jianghuang	MOL000953	CLR
Tianjihuang	MOL000211	Mairin
Tianjihuang	MOL000358	beta-sitosterol
Tianjihuang	MOL000359	sitosterol
Tianjihuang	MOL000422	kaempferol
Tianjihuang	MOL006772	poriferasterol monoglucoside_qt
Tianjih	MOL007	Tetramethoxyluteolin

uang	879	
Tianjihuang	MOL007880	3,5,7,3′,5′ pentahydroxy flavonol
Tianjihuang	MOL000098	quercetin
Xiakucao	MOL000358	beta-sitosterol
Xiakucao	MOL000422	kaempferol
Xiakucao	MOL004355	Spinasterol
Xiakucao	MOL000449	Stigmasterol
Xiakucao	MOL004798	delphinidin
Xiakucao	MOL000006	luteolin
Xiakucao	MOL006767	Vulgaxanthin-I
Xiakucao	MOL006772	poriferasterol monoglucoside_qt
Xiakucao	MOL006774	stigmast-7-enol
Xiakucao	MOL000737	morin
Xiakucao	MOL000098	quercetin
Yanhusuo	MOL001454	berberine
Yanhusuo	MOL001458	coptisine
Yanhusuo	MOL001460	Cryptopin
Yanhusuo	MOL001461	Dihydrochelerythrine
Yanhusuo	MOL001463	Dihydrosanguinarine
Yanhusuo	MOL001474	sanguinarine
Yanhusuo	MOL000217	(S)-Scoulerine
Yanhusuo	MOL002670	Cavidine
Yanhu	MOL002	(R)-Canadine

suo	903	
Yanhusuo	MOL000359	sitosterol
Yanhusuo	MOL004071	Hyndarin
Yanhusuo	MOL004190	(-)-alpha-N-methylcanadine
Yanhusuo	MOL004191	Capaurine
Yanhusuo	MOL004193	Clarkeanidine
Yanhusuo	MOL004195	CORYDALINE
Yanhusuo	MOL004196	Corydalmine
Yanhusuo	MOL004197	Corydine
Yanhusuo	MOL004198	18797-79-0
Yanhusuo	MOL004199	Corynoloxine
Yanhusuo	MOL004200	methyl-[2-(3,4,6,7-tetramethoxy-1-phenanthryl)ethyl]amine
Yanhusuo	MOL004202	dehydrocavidine
Yanhusuo	MOL004203	Dehydrocorybulbine
Yanhusuo	MOL004204	dehydrocorydaline
Yanhusuo	MOL004205	Dehydrocorydalmine
Yanhusuo	MOL004208	demethylcorydalmatine
Yanhusuo	MOL004209	13-methyldehydrocorydalmine
Yanhusuo	MOL004210	(1S,8'R)-6,7-dimethoxy-2-methylspiro[3,4-dihydroisoquinoline-1,7'-6,8-dihydrocyclopenta[g][1,3]benzodioxole]-8'-ol
Yanhusuo	MOL004763	Izoteolin
Yanhusuo	MOL004214	isocorybulbine
Yanhusuo	MOL004215	leonticine
Yanhu	MOL004	13-methylpalmatrubine

suo	216	
Yanhusuo	MOL004220	N-methyllaurotetanine
Yanhusuo	MOL004221	norglaucing
Yanhusuo	MOL004224	pontevedrine
Yanhusuo	MOL004225	pseudocoptisine
Yanhusuo	MOL004226	24240-05-9
Yanhusuo	MOL004228	saulatine
Yanhusuo	MOL004230	stylopine
Yanhusuo	MOL004231	Tetrahydrocorysamine
Yanhusuo	MOL004232	tetrahydroprotopapaverine
Yanhusuo	MOL004233	ST057701
Yanhusuo	MOL004234	2,3,9,10-tetramethoxy-13-methyl-5,6-dihydroisoquinolino[2,1-b]isoquinolin-8-one
Yanhusuo	MOL000449	Stigmasterol
Yanhusuo	MOL000785	palmatine
Yanhusuo	MOL000787	Fumarine
Yanhusuo	MOL000790	Isocorypalmine
Yanhusuo	MOL000791	bicuculline
Yanhusuo	MOL000793	C09367
Yanhusuo	MOL000098	quercetin
Yinchen	MOL000354	isorhamnetin
Yinchen	MOL000358	beta-sitosterol
Yinchen	MOL004609	Areapillin
Yinche	MOL005	Genkwanin

n	573	
Yinchen	MOL007274	Skrofulein
Yinchen	MOL008039	Isoarcapillin
Yinchen	MOL008040	Eupalitin
Yinchen	MOL008041	Eupatolitin
Yinchen	MOL008043	capillarisin
Yinchen	MOL008045	4'-Methylcapillarisin
Yinchen	MOL008046	Demethoxycapillarisin
Yinchen	MOL008047	Artepillin A
Yinchen	MOL000098	quercetin
