# Supplementary material for: Systems Pharmacology-Based Strategy to Investigate the Mechanism of Ruangan Lidan Decoction for Treatment of Hepatocellular Carcinoma
Source: Comput Math Methods Med. 2022 Dec 17;2022:2940654. doi: 10.1155/2022/2940654 (PMC9791079; doi:10.1155/2022/2940654)
Supplement: Supplementary 2 — Supplementary Table S2: the detailed information of the 170 disease genes. [file 2940654.f2.pdf]

Gene Symbol	Gene ID
CTNNB1	1499
TP53	7157
AXIN1	8312
PIK3CA	5290
TSC2	7249
TSC1	7248
CASP8	841
EGF	1950
PDGFRL	5157
AFP	174
SLC5A5	6528
MET	4233
APC	324
PTEN	5728
KRAS	3845
CDKN2A	1029
EGFR	1956
TERT	7015
BRAF	673
CDH1	999
ERBB2	2064
AKT1	207
HRAS	3265
RET	5979
MSH2	4436
BRCA1	672
IGF2R	3482
MTOR	2475
HNF1A	6927
CCND1	595
MLH1	4292
ATM	472
MSH6	2956
VHL	7428
NRAS	4893
CDC73	79577
RB1	5925
BRCA2	675
CDKN3	1033
PTCH1	5727
SMAD4	4089
FHIT	2272
MTUS1	57509

LRRC56	115399
TGFBR2	7048
EPCAM	4072
FGFR3	2261
FLCN	201163
PMS2	5395
CHEK2	11200
VEGFA	7422
FASLG	356
HNF1B	6928
ESR1	2099
STK11	6794
PALB2	79728
CDKN1A	1026
HULC	728655
NKX2-1	7080
BAX	64114
SETD2	29072
MYC	57008
CDKN1B	1027
FGFR2	2263
OGG1	4968
KRT7	9119
MDM2	27085
STAT3	6774
MUC1	10071
HIF1A	55662
TNFRSF10B	111255642
MAP2K1	5604
IGF2	10644
MUTYH	4595
HEIH	100859930
HEPN1	641654
PTGS2	103752588
KIT	700
NTRK1	4914
PBRM1	55193
MMP9	109729184
FAS	355
SMARCA4	6597
BCL2	7917
KRT19	160313
FLT4	2324
KDR	3791

RAF1	5894
PDGFRB	5159
FLT3	84922
FLT1	391533
TOP2A	7153
FKBP1A	100528031
ESR2	2100
IMPDH1	3614
HMGCR	3156
DNAJB1	3337
FGFR1	11116
IMPDH2	3615
PRKACA	5566
CD274	29126
PDGFRA	5156
NFE2L2	111365149
IDH1	3417
CREBBP	10847
FGFR4	109279841
IDH2	3418
PDCD1	5133
TYMS	7298
PPAT	100289640
TOP1	100874223
SF3B1	23451
PCDH9	100874064
GNAS	2778
OSMR	9180
DDR2	4921
CSF1R	111188152
ABL1	25
DPYD	1806
POLD1	5424
POLE	5426
TUBB2B	347733
ARID2	100506681
TUBB2A	7280
TUBB6	84617
TUBB3	100130187
TUBB4A	10382
TUBB	197331
TUBB4B	10383
TUBB8	121014
TUBB1	140004

AR	197358
ARID1A	8289
EPHA2	1969
CTLA4	1493
TP63	8626
CACNA2D1	781
PRIM1	5557
FBXW7	55294
NRG1	100856811
GOPC	57120
ERBB4	2066
PTPN13	5783
PREX2	80243
LRP1B	53353
CUL4A	100129008
GPC3	110806308
HMGA2	8091
TEK	27285
MAPK11	5600
ERBB3	2065
HPSE	10855
FRK	108281156
AXIN2	8313
ZFHX3	463
FGF2	2247
LEF1	51176
SSTR5	146336
SSTR2	6752
NOTCH1	101928483
FOXO3	2309
QKI	9444
MN1	196394
NDRG1	10397
LATS1	9113
PTPRT	11122
PTPN11	5781
ACVR1	130399
MYD88	4615
NOTCH2	4853
